# Luteal phase oral dexamethasone administration alters the endometrial steroid milieu

**DOI:** 10.1530/EC-24-0638

**Published:** 2025-04-09

**Authors:** Natalie Z M Homer, Moira Nicol, Mayank Madhra, Gregorio Naredo-Gonzalez, Sofia Laforest, Ov D Slayden, Stephen G Hillier, Brian R Walker, Pamela Warner, Ruth Andrew, Hilary O D Critchley

**Affiliations:** ^1^ Mass Spectrometry Core, Edinburgh Clinical Research Facility, and University/BHF Centre for Cardiovascular Science, University of Edinburgh, The Queen’s Medical Research Institute, Edinburgh, UK; ^2^ Centre for Reproductive Health, Institute for Regeneration and Repair, University of Edinburgh, Edinburgh, UK; ^3^ Simpson Centre for Reproductive Health, Royal Infirmary of Edinburgh, Edinburgh, UK; ^4^ Centre for Cardiovascular Science, University of Edinburgh, The Queen’s Medical Research Institute, Edinburgh, UK; ^5^ Oregon National Primate Research Center, Beaverton, Oregon, USA; ^6^ Translational and Clinical Research Institute, Newcastle University, Newcastle, UK; ^7^ Centre for Cardiovascular Science, University of Edinburgh, Edinburgh, UK; ^8^ Usher Institute of Population Health Sciences & Informatics, University of Edinburgh, Edinburgh, UK

**Keywords:** endometrium, menstruation, luteal phase, low dose dexamethasone, glucocorticoids, sex steroids, liquid chromatography tandem mass spectrometry (LC-MS/MS)

## Abstract

We previously published the DexFEM trial, which showed that in women with heavy menstrual bleeding (HMB), oral dexamethasone reduces menstrual blood loss. Here, we report a pharmacodynamic analysis exploring the likely mechanism for this effect. We studied oral dosing with dexamethasone during the mid-luteal phase of two menstrual cycles (1.5 mg daily, 5 days) in five women with HMB (six recruited aged 41–50 years, one withdrew before treatment). Steroid hormones were profiled in serum and endometrium by liquid chromatography-tandem mass spectrometry (LC-MS/MS). We found that following oral dosing, dexamethasone reached the endometrium and that, compared to the preceding control cycle, cortisol (active), cortisone (inactive) and intermediate 11-deoxycortisol were reduced in all samples assessed, both endometrial (*n* = 4) and serum (*n* = 5). Concentrations of androgens, androstenedione and testosterone were reduced in serum but not in all tissue samples. This proof-of-concept pharmacodynamic study supports the inference that dexamethasone is effective in HMB by altering endometrial glucocorticoid concentrations.

**Significance statement:**

New treatments are needed for HMB, a debilitating symptom experienced by many women. The association previously found between local endometrial glucocorticoid deficiency and HMB stimulated research into the role for glucocorticoids in the physiological process of menstruation. We report a proof-of-concept experimental medicine study profiling steroids in endometrial biopsies, demonstrating that dexamethasone administered orally penetrates the endometrium and is associated with changes in circulating endogenous steroid profiles (glucocorticoids and androgens) and in glucocorticoids in the endometrium. Dexamethasone thus has potential utility to modify glucocorticoid profiles and endometrial steroids in those with HMB. These findings augment the results of the recently reported adaptive clinical trial we conducted, also in women with HMB, showing that oral dexamethasone reduces menstrual blood loss.

## Introduction

The common symptom of heavy menstrual bleeding (HMB), experienced by at least one in three women, has an adverse impact on quality of life and productivity. HMB is under-recognised and under-researched (1, 2). Current treatment options are not patient-specific and many find current medical therapy for HMB has intolerable side effects, leading to cessation of use (1). Indeed, there has not been a ‘new class’ of medical approaches for HMB since the levonorgestrel-releasing intrauterine system was licensed in 2001. Dissecting the mechanistic pathways involved in HMB is an essential step in the development of targeted, effective and more personalised medical treatment strategies for this debilitating complaint.

Many lines of evidence underpin menstruation as an inflammatory event (3). The endometrium is exposed to an orchestrated sequence of repetitive events of inflammation (injury) and repair consequent upon sequential exposure to 17β-oestradiol, progesterone, followed by progesterone withdrawal as a consequence of corpus luteum regression (3). Progesterone withdrawal is the trigger for the cascade of events that culminates in shedding of the upper zone of the endometrium (menstruation) (3, 4, 5). At the time of progesterone withdrawal, the endometrium has an increased expression and generation of potent vasoactive prostaglandins (PGs), namely PGE2 (vasodilator) and PGF2α (vasoconstrictor), with vasoconstriction of spiral arterioles in the functional zones of the endometrium (3).

In addition to the well-recognised roles for 17β-oestradiol, progesterone and prostaglandins, glucocorticoids play an important role in modulating these processes. Endometrial immune cells express only glucocorticoid receptors, and stromal and vascular cells express both progesterone and glucocorticoid receptors (6, 7, 8, 9, 10). Concentrations of cortisol, the principal human glucocorticoid, in tissues, including the human endometrium, are influenced not only by their systemic levels but also by local interconversion of cortisol with its inert metabolite, cortisone, catalysed by the isozymes of 11β-hydroxysteroid dehydrogenases (11βHSDs). Local generation of cortisol within many tissues by 11βHSD1 is important for limiting inflammatory responses and potentiating vasoconstriction (9). We have shown that women with HMB have excess local inactivation of cortisol to cortisone by 11βHSD2 (7) accompanied by greater endometrial biosynthesis of prostaglandins (11). Glucocorticoids modulate prostaglandin production (12), so these changes in glucocorticoids provide a cogent explanation for the increased PGE2:PGF2α ratio (and relative vasoconstrictor deficiency) in endometrium from women with HMB (13, 14, 15) and suggest a potential deficiency of cortisol could underpin impaired anti-inflammatory repair mechanisms.

We therefore hypothesised that local endometrial glucocorticoid deficiency and consequent changes in prostaglandin production contribute to deficient vasoconstriction of the endometrial vasculature and increased menstrual bleeding. Furthermore, rescuing local glucocorticoid deficiency in the endometrium with oral dexamethasone administration in the luteal phase might provide a novel therapeutic strategy for women with HMB. We undertook an adaptive clinical trial in women with HMB and demonstrated that oral dexamethasone (1.8 mg daily) reduced menstrual blood loss (16), however, the mechanism of this effect could be local or via other systemic actions of dexamethasone.

In an experimental medicine study undertaken before the trial to explore the local effects of oral dexamethasone within the endometrium, we collected serum and endometrial tissue samples. We have now applied multi-steroid profiling by liquid chromatography-tandem mass spectrometry (LC-MS/MS) to address the following questions: i) is dexamethasone detectable in the endometrium after two cycles of oral administration?; ii) how does the circulating and endometrial endogenous steroid milieu after oral dexamethasone administration differ from the preceding untreated cycle?

## Methods

### Study design and participants

Participants in this proof-of-concept, unblinded within-subject study were six women (aged between 41 and 50 years) referred to gynaecology services in NHS Lothian, Scotland, UK, with symptoms of HMB.

Inclusion/exclusion criteria for this study are available in the published protocol for the subsequent response-adaptive randomised placebo-controlled dose-finding parallel-group trial (DexFEM) (17). Inclusion criteria included participants reporting regular menstrual cycles every 21–42 days. Although the age inclusion criterion was 18–45 years, those consulting for HMB and agreeing to participate were women over 40 years (two aged 41 years, three aged 45 years and one aged 50 years – one 45 years-old participant later withdrawing before treatment). Exclusion criteria included use of concurrent steroids, i.e., glucocorticoid treatment (low-potency topical steroids allowed) or sex steroid administration by any route in the previous 1 month (16). The study received ethics approval from Lothian Research Ethics Committee (10/S1402/59) and all participants gave written informed consent.

This study involved three menstrual cycles, first an untreated ‘control’ menstrual cycle, then two consecutive cycles in which participants received 0.75 mg oral dexamethasone twice daily (Tayside Pharmaceuticals, UK) for 5 days in the mid-luteal phase. One participant withdrew after the control cycle. All assessments were made during the late luteal phase of participants’ cycles, i.e., 1–5 days before the start of the next menstrual period (Table 1).

**Table 1:** Participant-specific sampling times of blood and endometrium.

Study participant		Time of biopsy	Time of blood sample	Serum E2 (pmol/L)	Serum P4 (nmol/L)	Histology	Start of next menses relative to date of biopsy (days after)
S1	Control cycle	18:00	16:30	155	4.0	Late secretory	+2
S2	Control cycle	17:00	12:40	354	33.1	Late secretory	+3
S3	Control cycle	11:00	12:30	304	19.8	Late secretory	+2
S4	Control cycle	11:15	09:30	586	20.5	Late secretory	+3
S5	Control cycle	10:50	09:30	361	10.1	Late secretory	+2
S6	Control cycle	13:00	12:10	252	13.6	Late secretory	+1
S1	2nd treatment cycle	11:45	10:20	133	4.9	Late secretory	+2
S2	2nd treatment cycle	13:50	18:30	273	46.2	Late secretory	+2
S3	2nd treatment cycle	13:45	12:00	238	8.2	Late secretory	+1
S4	2nd treatment cycle	14:00	12:30	672	28.3	Late secretory	+3
S5	2nd treatment cycle	14:40	13:30	744	8.9	Late secretory	+5
S6*	2nd treatment cycle	NA	NA	NA	NA	No biopsy	NA

* Participant withdrew; NA, not applicable.

Participant (numbered S1-6) specific sampling times of blood and the endometrium, i.e., before and following oral dexamethasone administration. Table includes circulating 17β–oestradiol (E2) and progesterone (P4) levels measured by immunoassay at the time of endometrial and blood collection, overall histological dating of the endometrium and the number of days after endometrial sampling that the next menses started. Withdrawal of one participant (S6) before the treatment cycle meant 11 serum and tissue samples were collected. However, S6’s (single) control tissue sample was not analysed, and S4’s dexamethasone-treated tissue sample was insufficient for analysis (<100 mg), so nine tissue samples were assayed.

### Participant assessments

Participants underwent: i) Endometrial sampling using a fine suction curette (endometrial Pipelle® sampler, Laboratoire CCD, France) in an outpatient setting in the late luteal phase of the control cycle and the 2nd dexamethasone-treated cycle. In the treated cycle, the biopsy took place between the 9th and 10th dexamethasone dose, on the 5th and final day of exposure. Tissue was stored at −80°C. ii) Blood sampling at both biopsy visits. Venous blood was drawn into an S-Monovette® 4.9 mL serum gel with Clotting Activator tube, which was immediately centrifuged (800 ***g***, room temperature, 15 min). The supernatant was withdrawn, and serum was transferred to a 2.5 mL microtube and frozen at −20°C.

For steroid profiling, dexamethasone and endogenous steroids were measured by LC-MS/MS in serum (18, 19), and in endometrial tissue (∼100 mg), following homogenisation and extraction (Supplementary methods (see section on Supplementary materials given at the end of the article)).

One participant withdrew before the treatment cycle; neither treatment sample was obtained, and her control tissue sample was excluded from analysis. The treatment tissue sample for another participant was of inadequate weight (<100 mg) for steroid analyses. We thus report pairs of samples (control plus treated) for five women regarding serum but for four women regarding endometrial tissue.

### Presentation of data

For maximum insight in this exploratory study, data values for each participant are plotted individually for control and intervention cycles, and we comment on findings for measures where all participants showed change in the same direction between no treatment and dexamethasone treatment. In supplemental methods we explain why formal statistical analyses are not reported.

## Results

All participants had symptoms of HMB. One participant withdrew before the treatment phase of the study. Results of 17β-oestradiol and progesterone concentrations in blood, collected at the time of biopsy and ascertained by rapid ELISA, were consistent with women being in the late luteal phase of their cycle, as was biopsy date relative to the start of the next menstrual bleeding (Table 1). In the 2nd consecutive dexamethasone treatment cycle, all participants demonstrated endometrial histological features consistent with exposure to luteal-phase concentrations of 17β-oestradiol and progesterone (Table 1). Serum progesterone (Fig. 2A) levels measured by LC-MS/MS correlated with immunoassay measures. Progesterone and 17β-oestradiol levels measured by immunoassay ELISA (Table 1) confirmed ovulatory activity.

**Figure 1:**
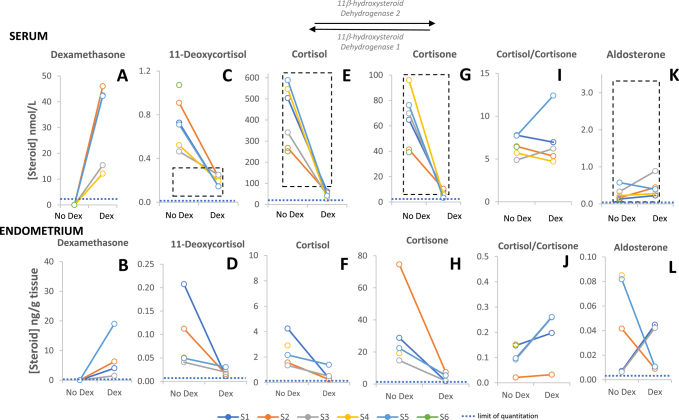
Circulating (serum) concentrations, alongside endometrium concentrations, of exogenous dexamethasone, endogenous glucocorticoid (cortisol, cortisone and 11-deoxycortisol), the mineralocorticoid aldosterone and the ratio of cortisol to cortisone (as an index of 11βHSD activity). (*n* = 11 serum, *n* = 9 tissue) Premenopausal reference ranges indicated by dashed lines in serum are from Mayo Clinic (https://www.mayocliniclabs.com/search?q=steroids). Limits of quantitation for each steroid are shown with dotted lines. The number of complete pairs of values (no dex and dex-treated, with joining line) for serum graphs A, C, E, G, I and K = 5 and for endometrium graphs B, D, F, H, J and L = 4. Dex, dexamethasone.

Dexamethasone was detected in samples obtained during treated cycles, in both serum (Fig. 1A) and endometrium (Fig. 1B), but not in control cycle samples. Following treatment, cortisol, cortisone and the intermediate 11-deoxycortisol were seen to decrease markedly in all participants assessed, in serum and endometrium (Fig. 1C, D, E, F, G, H). Cortisol was present in serum in higher amounts than cortisone in both control and dexamethasone-treated cycles (Fig. 1I shows all ratios greater than 1), whereas in the endometrium the reverse was found (Fig. 1J shows all ratios less than 1). While in serum the calculated ratio of cortisol to cortisone did not change consistently after treatment (Fig. 1I), in endometrial tissue these ratios increased (Fig. 1J). 11-Deoxycortisol in serum in the control cycle was higher in these HMB subjects than the typical reference range (Fig. 1C).

The direction of change from no treatment to dexamethasone treated in the amounts of the mineralocorticoid aldosterone was inconsistent in serum and, particularly, endometrium (Fig. 1K and L).

**Figure 2:**
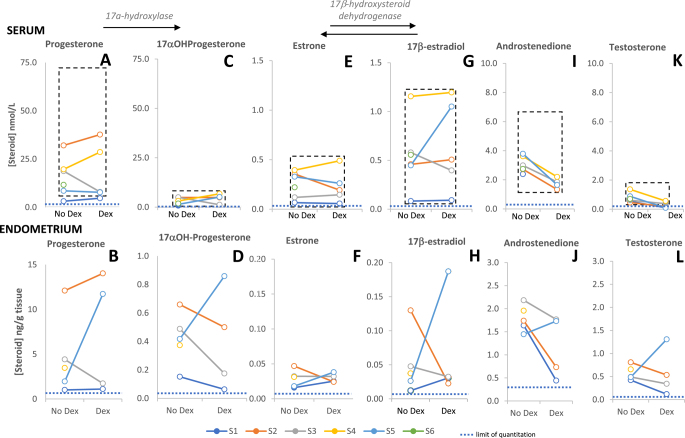
Circulating (serum) progestogens, oestrogens and androgens during the luteal phase of the menstrual cycle, alongside their endometrial tissue levels. Premenopausal reference ranges shown with dashed lines in serum are from Mayo Clinic (https://www.mayocliniclabs.com/search?q=steroids). Limits of quantitation for each steroid are shown with dotted lines. The number of complete pairs of values (no dex and dex-treated, with joining line) for serum graphs A, C, E, G, I and K = 5, 4, 5, 5, 4 and 5 respectively, and for endometrium graphs B, D, F, H, J and L = 4. Dex, dexamethasone.

Comparing between control and dexamethasone-treated cycles, no consistent change was observed in progesterone or oestrogens in serum or endometrium: progesterone (Fig. 2A and B), 17α-hydroxyprogesterone (Fig. 2C and D) and oestrogens (Fig. 2E, F, G, H).

Androgens (androstenedione, testosterone) were lower in serum following treatment (Fig. 2I and K), whereas in the endometrium, in three out of four participants, levels fell and in one participant rose (S5) (Fig. 2J and L). Circulating dehydroepiandrosterone sulphate was lower in serum following treatment (Table 2), while the 11oxoandrogen, 11β-hydroxyandrostenedione, was detected in circulation before but not following treatment (Table 2). These steroids were not measured in tissue due to insufficient tissue remaining for the extraction protocol required. Amounts of 5α-dihydrotestosterone (DHT) were close to the lower limits of the method and detected in only 50% of the samples from untreated cycles (data not shown).

**Table 2:** Concentrations of 11β-hydroxyandrostenedione (11OHA4) and dehydroepiandrosterone sulphate (DHEAS) measured in serum by liquid chromatography-tandem mass spectrometry.

Study participant	(11OHA4) nmol/L		(DHEAS) mmol/L	
	Control cycle	2nd dexamethasone treatment cycle	Control cycle	2nd dexamethasone cycle
S1	2.07	<LLOQ	2.54	0.49
S2	2.03	<LLOQ	1.89	0.71
S3	1.21	<LLOQ	1.60	0.64
S4	1.89	<LLOQ	3.40	0.82
S5	4.44	<LLOQ	2.85	0.53
S6	1.39	NA	2.47	NA

NA, patient withdrew before treatment phase; LLOQ, lower limit of quantification.; 11OHA4, 11β-hydroxyandrostenedione; DHEAS, dehydroepiandrosterone sulphate.

## Discussion

HMB is a common debilitating and chronic complaint. Treatment options are limited and often involve invasive fertility-ending surgery (20). Medical interventions may be more acceptable to manage HMB, and in this setting, rescue of putative endometrial glucocorticoid deficiency with a short course of oral low-dose dexamethasone administered in the luteal phase has been demonstrated to reduce menstrual blood loss (7, 16). In this study, the glucocorticoid dexamethasone was detected in the endometrium following systemic (oral) administration for 5 days in the luteal phase of the menstrual cycle. Our findings suggest that dexamethasone penetration of the endometrium is associated with changes to the endometrial steroid milieu, which may contribute to the effects demonstrated in our adaptive trial where short-term luteal-phase treatment with dexamethasone showed amelioration of symptoms of HMB (16). The fact that systemic dexamethasone treatment was associated with suppression of circulating cortisol, with the potential for side effects, suggests that a possible future therapeutic strategy to minimise these could be direct/local administration. Further research is required concerning optimal regimens and route(s) for dexamethasone delivery.

The main circulating endogenous glucocorticoid in humans is cortisol, and a major route of inactivation in the endometrium is via 11βHSD2, generating inert cortisone. In serum, the cortisol to cortisone ratio typically exceeds five (21), as observed in this current study (Fig. 1I), although a large proportion (approx. 90%) of cortisol is protein-bound, e.g., cortisol-binding globulin and albumin, and not available to penetrate the tissue (22). We have previously documented the cell-specific endometrial expression of 11βHSDs and variation across the normal menstrual cycle. In particular, endometrial expression of 11βHSD1 and expression of the glucocorticoid receptor are increased at the time of normal menses, consistent with a role for increased local cortisol concentrations (23) limiting the inflammatory response and potentiating vasoconstriction (9). Measurements of steroid quantities in endometrial tissue to support this concept, however, are rarely available in healthy subjects throughout the menstrual cycle due to the invasive nature of endometrial biopsy. Häkkinen *et al.* (24, 25) previously measured cortisol and cortisone in eutopic endometrial tissue in women with endometriosis undergoing laparoscopic tubal ligation, reporting a 0.40 cortisol/cortisone ratio. In our study, we have used current gold-standard LC-MS/MS steroid profiling methodology to quantify cortisol and cortisone in the endometrial tissue of women with HMB and found control cycle cortisol to cortisone ratios between 0.02 and 0.15 (Fig. 1J) in this setting, all considerably lower than the 0.4 average reported by Häkkinen (24, 25). This balance towards cortisone is likely to be the consequence of enzymatic activity and unlikely to be due to only the free pool of cortisol entering the tissue. Indeed, the amounts of free cortisol and cortisone are similar in circulation (20). While cortisol to cortisone ratios all increased after oral dexamethasone treatment, they remained in the range 0.03–0.26 (Fig. 1J). This increase may be due to competition for inactivation by 11βHSD2 (26).

Dexamethasone is a steroid commonly used for its anti-inflammatory properties and a potent cortisol surrogate and glucocorticoid receptor agonist. It is usually administered orally, and here we addressed the question of whether it was mediating its effects by gaining access to the endometrium. With LC-MS/MS methodology, we confirmed, through measurements in serum, compliance of the subjects taking oral dexamethasone and furthermore, that oral dexamethasone administration results in penetration of the drug into endometrial tissue, where it may exert beneficial effects. The amounts achieved in endometrial biopsies were in the order of 1.49–19.02 ng/g which roughly equates to 50 pmol/g. If we assume a gram of tissue is equivalent to 1 mL in volume, this is within the effective range for dexamethasone, estimating a *K*_D_ binding to the glucocorticoid receptor of 10 nmol/L.

Systemic administration of dexamethasone would be expected to cause hypothalamic–pituitary–adrenal (HPA) suppression, even in the dose (1.5 mg/day) used in this current study, and indeed cortisol concentrations in the blood of patients were substantially suppressed, as well as those in the endometrium. Adrenal suppression following dexamethasone treatment was also evident, with very low 11-deoxycortisol, an intermediate between 17α-hydroxyprogesterone and cortisol, in the circulation (Fig. 1C) and in endometrial tissue (Fig. 1D), and lowered androgens. If this therapeutic strategy is explored further, then local drug delivery, with, for example, novel vaginal or intrauterine drug delivery systems, may be preferable to minimise any potential systemic side effects. Nonetheless, the principle of delivery of dexamethasone to the endometrium having potential beneficial effects to the endometrial endocrine milieu has been established. Dexamethasone did not alter the amounts of oestrogen or progesterone in endometrial tissue. Oestrogens and progesterone play key roles in endometrial function (27). Androstenedione and testosterone were lowered in tissue for three of four participants, as well as in blood for all women, during the dexamethasone-treated cycle. This is likely secondary to suppression of the HPA axis and may contribute to the beneficial effects on HMB, given the role androgens may exert in the context of endometrial function, such as in regulation of endometrial cell proliferation (28).

The strengths of this study are the paired study design and the use of gold-standard LC-MS/MS methodology (29) to profile multiple steroids in carefully characterised endometrial samples from women with the complaint of HMB before and during treatment with oral dexamethasone, in order to better understand the mechanism of action of a potential novel intervention for the management of HMB.

A limitation is that of six participants recruited, one withdrew before treatment, and her control cycle tissue sample also was not included in steroid analysis, and one participant’s tissue sample in their dexamethasone-treated cycle was of insufficient weight for analysis. Conventional hypothesis-testing analysis was thus ruled out on account of lack of power. We have therefore plotted all individual data points so readers can peruse our data alongside our account of our findings. A second limitation of the study is the lack of reference ranges on endometrial tissue for cortisol and cortisone in healthy women who do not experience HMB. A comparative study with women who have normal and, by set criteria, heavy-measured menstrual blood losses would address this concern.

In summary, oral administration of dexamethasone can reach the target tissue, endometrium, and modify tissue steroid profiles (notably cortisol and cortisone), and can potentially reverse endometrial glucocorticoid deficiency in women with HMB. We acknowledge that other common disorders affecting the HPA axis, such as polycystic ovarian syndrome, may have downstream effects on menstrual bleeding, which are worthy of exploration (30).

Our currently presented data complement our recent findings that a short 5-day course of oral dexamethasone reduced menstrual blood loss and provides further endorsement that a role for dexamethasone as a management strategy for HMB warrants further study (16). If pursued further for clinical management of HMB, then local administration (vaginal/intrauterine delivery) may be preferable to avoid potential undesirable systemic side effects. Local delivery would need to transfer steroid to the endometrium adequately and may result in a smaller systemic effect than observed with oral dexamethasone, and thus be more acceptable. The unmet need for targeted medical treatment approaches for this debilitating complaint remains ever important.

## Supplementary materials



## Declaration of interest

HODC has received clinical research support for laboratory consumables and staff from Bayer AG, and has provided advisory board/consultancy advice (all paid to the institution) for Bayer AG, PregLem SA, Gedeon Richter, Vifor Pharma UK Ltd, AbbVie Inc, Myovant Sciences GmbH, Theramex and BII (BioInnovation Institute), Copenhagen. HODC has received royalties from UpToDate for an article on abnormal uterine bleeding. Other authors have no conflicts of interest to declare.

## Funding

This work was supported by the UK Medical Research Councilhttps://doi.org/10.13039/501100000265 DCS/DPFS funding scheme grant number: MR/J003611/1 and MRC Centre Grant: MR/N022556/1. BRW is grateful for support from the British Heart Foundationhttps://doi.org/10.13039/501100000274 (RG11/4/28734) and the Wellcome Trusthttps://doi.org/10.13039/100010269 (107049/Z/15/Z).

## Author contribution statement

HODC, SGH, BRW, PW and OS conceptualised the study. PW, HODC, MN, GN-G, NZMH, RA and MM curated the data. HODC, MM, GN-G, SL, NZM, RA and PW conducted the formal analysis. HODC, PW and BRW secured funding for the research. NZMH and RA carried out the investigation. HODC, PW, MM, MN, GN-G, SL, NZMH and RA developed the methodology. NZMH and RA provided resources. HODC, RA and PW supervised the work. NZMH, SL and RA validated the finding. HODC, NZMH, RA and PW drafted the original manuscript. All authors contributed to reviewing and editing the final version.

## Data availability

The data underlying this article are available upon request in Edinburgh Data Share.
