# Supplementary material for: Luteal phase oral dexamethasone administration alters the endometrial steroid milieu
Source: Endocr Connect. 2025 Apr 9;14(5):e240638. doi: 10.1530/EC-24-0638 (PMC12007876; doi:10.1530/EC-24-0638)
Supplement: Supplementary file 1 [file supplementary_materials.pdf]

## Supplemental Methods

### Statistical considerations

For a modest paired study design the non-parametric Wilcoxon matched pairs Signed Ranks test is generally used to compare (paired) measures between treated and control cycles. For this test it is the case that a minimum of six pairs is required to allow any statement of statistical significance at 5% level, and this can happen only if *all changes* are in the same direction. Of more concern, the p value returned for such analyses will be the same *regardless* of how marked or how minimal the same-direction changes are (i.e., p=0.31). We thus felt that reflection on findings should be based on perusal of data plots rather than potentially misleading p-values.

In the event, one of our participants withdrew before treatment, so we have a maximum of five pairs of change data. Furthermore, one woman's dexamethasone-treated tissue sample was insufficient for analysis (<100mg), so for tissue characteristics there are only four pairs of change data. This reduced n means that even if all pairs show change in the same direction, the p-value returned with 5 (or 4) pairs of data would be 0.063 (or 0.125).

Therefore, to avoid potential confusion between the lack of statistical power of analyses possible on data available, and *degree* of any effect of dexamethasone that might be shown, we decided *not* to conduct/report (underpowered) Wilcoxon analyses for our measures. Instead, we have plotted all individual data points (with change lines where both control and treated cycle measurements were made) so that readers can peruse changes observed, and reflect on our novel data in the light of their own endocrine knowledge.

That said, for any plot showing all changes in the same direction, formal analysis would have returned a p value of 0.063 if it involved 5 pairs (e.g., Figure 1E showing results for cortisol in serum), while if only 4 pairs were involved would return p=0.125 (e.g., Figure 2I for androstenedione in serum).

### LC-MS/MS measurement of steroids

### Measurement of multiple endogenous steroids in serum

Endogenous unconjugated steroids were profiled on an Acquity I-Class UPLC system and QTrap 6500+ mass spectrometer (AB Sciex, UK), based on our published LC-MS/MS steroid

profiling method(18)' (19). Serum (200 μL) was enriched with a mixture of isotopically labelled internal standards and extracted on an Extrahera liquid handling robot (Biotage, Sweden) alongside calibration curves of multiple endogenous steroids (0.0025 – 100 ng) following transfer into wells of a supported liquid extraction 96-well plate (ISOLUTE, SLE+ 400, Biotage, Uppsala, Sweden). Steroids in standards and samples were eluted with dichloromethane/isopropanol (98:2 v/v). Extracts were reduced to dryness and reconstituted in water/methanol (70:30 v/v; 100 μL). The extracts were injected (20 μL) onto a Kinetex C18 (150 x 2.1 mm; 2.6 μm) column (Phenomenex, UK) and steroids were separated using a mobile phase system of water with 0.05 mM ammonium fluoride (solution A) fluoride and methanol with 0.05 mM ammonium fluoride (solution B), at a flow rate of 0.3 mL/min and a column temperature of 50$^{o}$C. A gradient elution was conducted over 16 minutes, starting at 50% B for 4 minutes, rising to 75% B over 5 minutes, then to 100% B over 1 minute, held for 2 minutes, then returning to 50% B over 0.1 minutes and equilibrating for 3.9 minutes. The solvent flow was diverted to waste from 0-2 minutes and 11-16 minutes. The eluate from the LC column was transferred to the QTrap 6500+ mass spectrometer. Multiple reaction monitoring (MRM) parameters for each analyte with precursor – product mass transitions were performed as described in Denham et al (18). Quantities of each steroid in the samples were calculated using Quantitate® software (AB Sciex, UK) which uses the peak area ratio of the steroid/internal standards and relates this to the calibration curve. Lower Limits of Quantitation (LLOQ) for each steroid in serum were determined by assessment of accuracy and precision for 6 measurements at the low end of the calibration curve for both inter and intra-assay. Resulting LLOQ was 5 ng/mL for cortisol, 0.25 ng/mL for cortisone, 0.1 ng/mL for androstenedione, 0.5 ng/mL for testosterone, 1.25 ng/mL for 5α-dihydrotestosterone, 1.25 ng/mL for DHEA, 0.25 ng/mL for progesterone, 0.5 ng/mL for 17α-hydroxyprogesterone,

0.0625 ng/mL for 11-deoxycortisol, 0.125 ng/mL for 11-deoxycorticosterone, 0.0625 ng/mL for 17β-estradiol, 0.025 ng/mL for estrone and 0.0625 ng/mL for aldosterone.

**Measurement of dehydroepiandrosterone sulphate (DHEAS) in serum**

Serum (100 µL) was enriched with [2,2,3,4,4]-$^{2}H_5$-dehydroepiandrosterone sulphate (d5-DHEAS; 0.5 ng; Sigma Aldrich/Cerilliant, Dorset, UK) and extracted alongside a calibration curve of DHEAS (0.1 – 50 ng) through a phospholipid depletion plate (PLD+) (Biotage, Uppsala, Sweden), reduced to dryness under nitrogen and reconstituted in water/methanol (100 µL; 70:30 v/v). The extract was injected (20 µL) onto a Waters UPLC system fitted with a Kinetex Biphenyl (100 x 2.1 mm; 2.6 µm) column (Phenomenex, UK). The column was maintained at 50$^{o}$C and used a mobile phase system of water with 0.05 mM ammonium fluoride (solution A) fluoride and methanol with 0.05 mM ammonium fluoride (solution B), with a flow rate of 0.3 mL/min and a gradient starting from 55%B, rising to 100%B and returning to 55%B over 20 minutes. Eluate was introduced into a QTrap 6500+ mass spectrometer for mass analysis in negative ion mode, where the MRM parameters for DHEAS and its internal standard d5-DHEAS were as follows: DHEAS (*m/z* 367.1→96.9, 79.9 (-110, -10, -38, -11 V and -35V)) and d5-DHEAS (*m/z* 372.1→97.9 (-40, -10, -36, -7 V)). Quantities of DHEAS (ng) in the samples were calculated using Quantitate® software using the peak area ratio of DHEAS/d5DHEAS. LLOQ was calculated as before, where LLOQ = 1 ng/mL DHEAS.

**Measurement of 11β-hydroxyandrostenedione and dexamethasone in serum**

Serum was enriched with [4,6α,21,21]-$^{2}H_4$-dexamethasone (d4-dex; 20 ng; CDN Isotopes, Quebec, Canada) and [2,3,4]-$^{13}C_3$-testosterone ($^{13}C_3$-T; 0.25 ng; Sigma-Aldrich/Cerilliant, UK). For 11β-hydroxyandrostenedione (11OHA4) a calibration curve with range 0.025- 10 ng/mL was prepared alongside a calibration curve of 0.5 – 100 ng/mL for dexamethasone. Serum (100 μL) was extracted by supported liquid extraction (ISOLUTE, SLE+ 200, Biotage AB Uppsala, Sweden), and extracts were injected (20 µL) onto an ACE 2 Excel C18-PFP (150 × 2.1 mm, 2 μm, ACT Technologies, Aberdeen, UK) column at 40°C, and separated, adapted (18). Briefly, at a flow rate of 0.4 mL/min, the chromatography conditions began with 90:10 water with 0.1% FA (solution A) and acetonitrile with 0.1% FA (solution B) which was maintained for 1 min, followed by a 12-min linear gradient to 50% B, maintained for 2 min, before returning to 10% B by 18 min, maintained for 3 min to re-equilibrate. The eluate from the LC column was transferred to a QTrap 6500+ instrument. MRM parameters of precursor – product mass transitions used for 11OHA4 ((*m/z* 303.1→121.0 (101, 10, 33, 16 V) and (*m/z* 303.1→145.0 (101, 10, 35, 18 V))) with retention time 9.3 min, measured alongside dexamethasone (*m/z* 393.1→373.2 (71, 10, 11, 16 V)), and isotopically labelled internal standard d4-dex (*m/z* 397.1→377.2 (51, 10, 11, 16 V)) with retention time 9.1 and 9.0 mins, respectively. Quantities of these steroids were calculated using MultiQuant® software (AB Sciex, UK) which uses the peak area ratio of the steroid/internal standards and relates this to the calibration curve. LLOQ was 0.025 ng/mL for 11OHA4 and 0.5 ng/mL for dexamethasone.

**Measurement of multiple steroids and dexamethasone in endometrial tissue**

Endometrial tissue (~100 mg, exact weight recorded) was homogenised in methanol (1 mL), reduced to dryness under nitrogen (60$^{o}$C) and re-suspended in 90:10 water/methanol (200 µL). The homogenate was enriched with isotopically labelled internal standards of

(endogenous steroids and d4-dex; 20 ng), transferred into wells of a supported liquid extraction plate (ISOLUTE, SLE+ 400, Biotage AB Uppsala, Sweden) and enriched with the isotopically labelled internal standard mix and d4-dex (2 ng) alongside a calibration standard curve (0.0025 – 25 ng). The sample was diluted with water (v/v, 0.1% formic acid; 200 µL) and eluted with 1.8 mL dichloromethane/propan-2-ol (v/v, 98:2). The eluent collected into a deep well collection plate (2 mL, Waters, Wilmslow, UK), reduced to dryness and reconstituted in 70:30 water/methanol (v/v, 100 µL) before being transferred to total recovery LC vials (Waters, UK).

Steroids in endometrial tissue homogenate extract were measured according to the endogenous steroid profiling LC-MS/MS method detailed above, with the addition of dexamethasone. Quantities of each steroid, calculated from the calibration curves, were divided by the mass of tissue per sample, to give the amount of steroid in ng/g tissue. LLOQ for steroids in tissue was calculated in ng/100 mg tissue; 0.05 ng/g for cortisol, testosterone, dexamethasone and cortisone, 0.5 ng/g for androstenedione, 0.125 ng/g for 5α-dihydrotestosterone, 17β-estradiol and 11-deoxycortisol, 0.25 ng/g for 17α-hydroxyprogesterone, 0.125 ng/g for progesterone and 0.063 ng/g for 11-deoxycorticosterone, estrone, dehydroepiandrosterone and aldosterone.
